# Aflibercept more effectively weans patients with neovascular age-related macular degeneration off therapy compared with bevacizumab

**DOI:** 10.1172/JCI159125

**Published:** 2023-01-17

**Authors:** Xuan Cao, Jaron Castillo Sanchez, Tapan P. Patel, Zhiyong Yang, Chuanyu Guo, Danyal Malik, Anuoluwapo Sopeyin, Silvia Montaner, Akrit Sodhi

**Affiliations:** 1 Wilmer Eye Institute, Johns Hopkins University School of Medicine, Baltimore, Maryland, USA.; 2 Department of Oncology and Diagnostic Sciences, School of Dentistry, Department of Pathology, School of Medicine, University of Maryland School of Dentistry, Baltimore, Maryland, USA; Marlene and Stewart Greenebaum Comprehensive Cancer Center, Baltimore, Maryland, USA.

**Keywords:** Ophthalmology, Therapeutics, Clinical practice, Drug therapy

## Abstract

**BACKGROUND:**

Studies assessing the efficacy of therapies for neovascular age-related macular degeneration (nvAMD) have demonstrated that aflibercept may have a longer treatment interval than its less-expensive alternative, bevacizumab. However, whether this benefit justifies the additional cost of aflibercept remains under debate. We have recently reported that a treat-and-extend-pause/monitor approach can be used to successfully wean 31% of patients with nvAMD off anti-VEGF therapy. Here, we examined whether the choice of therapy influences the outcomes of this approach.

**METHODS:**

In this retrospective analysis, 122 eyes of 106 patients with nvAMD underwent 3 consecutive monthly injections with either aflibercept (*n* = 70) or bevacizumab (*n* = 52), followed by a treat-and-extend protocol, in which the decision to extend the interval between treatments was based on visual acuity, clinical exam, and the presence or absence of fluid on optical coherence tomography. Eyes that remained stable 12 weeks from their prior treatment were given a 6-week trial of holding further treatment, followed by quarterly monitoring. Treatment was resumed for worsening vision, clinical exam, or optical coherence tomography findings.

**RESULTS:**

At the end of 1 year, eyes receiving bevacizumab had similar vision but required more injections (8.7 ± 0.3 treatments vs. 7.2 ± 0.3 treatments) compared with eyes receiving aflibercept. However, eyes treated with aflibercept were almost 3 times more likely to be weaned off treatment (43% vs. 15%) compared with eyes treated with bevacizumab at the end of 1 year.

**CONCLUSION:**

These observations expose an advantage of aflibercept over bevacizumab and have important clinical implications for the selection of therapy for patients with nvAMD.

**FUNDING:**

This work was supported by the National Eye Institute, NIH grants R01EY029750 and R01EY025705, Research to Prevent Blindness, the Alcon Young Investigator Award from the Alcon Research Institute, and the Branna and Irving Sisenwein Professorship in Ophthalmology.

## Introduction

Aflibercept (Eylea) and bevacizumab (Avastin) are the two most frequently used therapies for the treatment of neovascular age-related macular degeneration (nvAMD) (1). Both therapies target the vasoactive mediator VEGF. Aflibercept has been shown previously to have a longer treatment interval and may be more effective than bevacizumab for the treatment of some patients with nvAMD (2–4). However, whether this benefit justifies the additional expense of bimonthly aflibercept, at more than 10 times the cost of monthly bevacizumab, remains under debate. Both of these anti-VEGF therapies are typically administered indefinitely, raising concerns about the economic and social burden of frequent clinic visits for elderly patients (5).

Given the anticipated increase in the number of patients with nvAMD worldwide, the sustainability of indefinite intraocular injections with either therapy is unclear. Moreover, many patients are understandably hesitant to commit to a lifetime of monthly (or bimonthly) clinic visits and intraocular injections. This has motivated some clinicians to reduce the number of treatments and visits. This may help explain why the success of anti-VEGF therapies in real-world clinical practice has been less impressive than that observed in clinical trials (6). This has prompted examination of alternative approaches that refine initial treatment protocols without sacrificing the visual acuity benefits observed with monthly or bimonthly treatment.

One approach to reduction of the treatment burden is to monitor patients with nvAMD using a fixed interval but to only treat patients as needed or pro re nata (PRN). This approach can reduce the total annual number of injections but would not effect either the frequency of patient visits or the number of imaging studies performed. This reactive approach aims to capture most relapses promptly, while minimizing the number of treatments. An alternative approach to optimize the efficacy of a drug for each patient while minimizing the number of injections is the treat-and-extend (TAE) protocol, in which the response of an individual patient to treatment is used to determine whether the interval between treatments can be extended for that particular patient; this approach provides mandatory dosing but at a personalized schedule. TAE is a proactive approach that assumes that patients manifest a regular pattern of disease activity (i.e., a patient’s response to their previous injection can predict their response to a subsequent injection). TAE, therefore, can reduce annually both the total number of visits as well as the total number of injections. However, the TAE approach may still result in overtreatment of patients during the extension phase, and patients may be treated unnecessarily during the maintenance phase.

Both PRN and TAE approaches have been shown to be noninferior compared with monthly or bimonthly treatment with anti-VEGF therapy in multiple randomized multicentered clinical trials (7–10). We recently assessed a hybrid of the PRN and TAE approaches, which we termed treat-and-extend-pause/monitor (TEP/M), and demonstrated that this approach can be used to safely and effectively wean 31% of patients with nvAMD off anti-VEGF therapy in 1 year (11). Two other studies have also examined the efficacy of weaning patients with nvAMD off treatment (12, 13). However, the levels of success when weaning patients off treatment in these two studies were substantially different. In one study, 143 of 385 eyes (37%) were successfully weaned off treatment (13), while in the other study 100 of 598 eyes (17%) reached exit criteria (12). Explanations for these dissimilar results include differences in inclusion/exclusion criteria, treatment protocols, the time interval required to successfully wean patients off treatment, and the description of what defines a successful treatment pause; none of these criteria were clearly defined. We hypothesized that the variability in the success of weaning patients off treatment among these studies may be affected by the specific anti-VEGF agent used; whether the agent used could affect weaning was not explored by these prior studies. Here, we set out to determine whether the choice of anti-VEGF therapy (aflibercept or bevacizumab) influences the long-term outcomes for those patients who underwent treatment using the TEP/M approach.

## Results

### Patient demographics and baseline characteristics.

**Figure 1:**
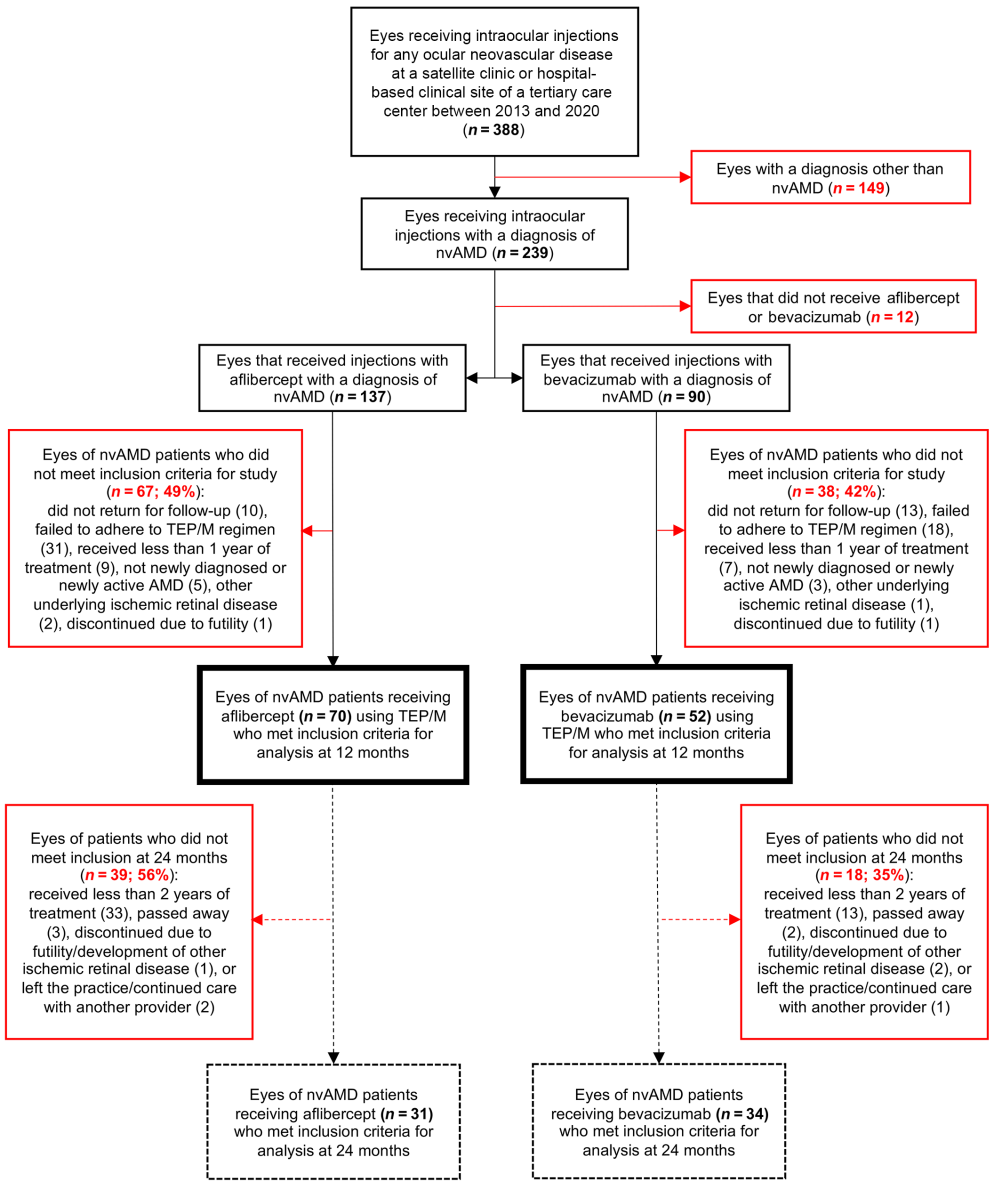
Flow diagram for identification of eligible eyes of patients with nvAMD who received treatment with either aflibercept or bevacizumab under the treat-and-extend-pause/monitor protocol. Primary endpoint at 12 months. Secondary endpoint at 24 months. TEP/M, treat-and-extend-pause/monitor.

Reviewing patient charts from the clinic of a single vitreoretinal surgeon at a tertiary care center from 2013 to 2020 identified 227 eyes of insured patients with a diagnosis of nvAMD who underwent intravitreal injections with either aflibercept or bevacizumab (Figure 1 and Table 1). One hundred and twenty-two eyes (106 patients) were eligible for the study based on the inclusion and exclusion criteria (see Methods for details). Seventy eyes (60 patients) received aflibercept and 52 eyes (46 patients) received bevacizumab. The choice of medication (aflibercept or bevacizumab) was made by the patient after they were informed of the potential benefits of each medicine, as previously described (14). The mean age (80.9 ± 0.8 yr vs. 78.7 ± 1.0 yr), presenting vision (56 ± 2 vs. 59 ± 3 Early Treatment Diabetic Retinopathy Study [ETDRS] letters), and presenting central subfield thickness (CST) on spectral-domain optical coherence tomography (SD-OCT) (311.4 ± 10.3 μm vs. 334.5 ± 15.6 μm) were similar for eyes of patients receiving aflibercept or bevacizumab, respectively. The median interquartile follow up was 28.5 months (Q1 of 18 months and Q3 of 43.5 months), with an interquartile range of 25.5 months. Sixty-five eyes (31 receiving aflibercept and 34 receiving bevacizumab) remained eligible through year 2.

### Outcome measurements after 3 mandatory intravitreal injections.

All patients received mandatory monthly treatment with aflibercept or bevacizumab for their first 3 visits. Visual and clinical exams and SD-OCT were performed at all clinic visits. After 3 consecutive monthly treatments, there was no difference in the reduction in CST on SD-OCT in eyes of patients treated with aflibercept compared with eyes of patients treated with bevacizumab (–71.3 ± 8.6 μm vs. –69.2 ± 17.7 μm; *P* = 0.337) 4–6 weeks following their third treatment (Table 2). The mean change in vision was similar in eyes of patients receiving aflibercept compared with eyes of patients receiving bevacizumab (2 ± 2 ETDRS letters vs. 2 ± 6 ETDRS letters). There was a higher percentage of patients with nvAMD with a mean improvement in vision of 5 letters or greater (49% vs. 33%, *P* = 0.021) but a similar mean decline in vision of 5 letters or greater (19% vs. 15%, *P* = 0.452) in patients receiving aflibercept compared with those receiving bevacizumab, respectively (Table 2).

### Outcome measurements at 6 months during the dynamic phase of the TEP/M protocol.

After the third treatment, the interval between treatments for patients with an improvement in vision, resolution (or near resolution) of fluid, and no hemorrhage was extended by 2 weeks (i.e., using the TEP/M protocol), as has previously been described (11). Six months after initiation of this protocol, the mean interval between treatments was longer for patients receiving aflibercept than for patients receiving bevacizumab (8.4 ± 0.3 weeks vs. 6.9 ± 0.4 weeks; *P* = 0.008; Table 3). In turn, the mean number of treatments for patients receiving aflibercept at month 6 was slightly lower than for patients receiving bevacizumab (4.8 ± 0.1 vs. 5.2 ± 0.1; *P* = 0.006). The mean change in CST (–72.1 ± 8.8 μm vs. –78.4 ± 14.7 μm; *P* = 0.469) and vision (4 ± 2 ETDRS letters vs. 0 ± 2 ETDRS letters; *P* = 0.172) was similar in patients receiving aflibercept compared with patients receiving bevacizumab at the end of month 6 (Table 3). There was also a similar percentage of patients with nvAMD with a mean improvement in vision of 5 letters or greater (51% vs. 46%) or a mean decline in vision of 5 letters or greater (19% vs. 27%) in patients receiving aflibercept compared with those receiving bevacizumab. However, the mean decline in vision of 10 or 15 letters or greater was lower in patients receiving aflibercept compared with those receiving bevacizumab (7.1% vs. 19%, *P* = 0.012) by month 6 (Table 3).

### Outcome measurements at 12 and 24 months during the steady-state phase of the TEP/M protocol.

The mean interval between treatments (using a maximal interval [cap] of 6 months for patients who were weaned off treatment) was longer for patients receiving aflibercept than for patients receiving bevacizumab (13.1 ± 0.9 weeks vs. 9.1 ± 0.8 weeks; *P* = 0.001) by the end of year 1 (Table 4). Accordingly, the mean number of treatments received was lower for patients receiving aflibercept than for patients receiving bevacizumab (7.2 ± 0.3 vs. 8.7 ± 0.3; *P* = 0.002). Compared with traditional bimonthly treatment with aflibercept (following 3 initial monthly treatments), the number of treatments using the TEP/M protocol was reduced by 10% (from 8.0 to 7.2). Compared with traditional monthly treatment with bevacizumab, the number of treatments using the TEP/M protocol was reduced by 33% (from 13.0 to 8.7) at 12 months (Table 4). The mean change in CST (–75.6 ± 9.7 μm vs. –71.5 ± 15.5 μm; *P* = 0.312) and vision (1 ± 2 ETDRS letters vs. 4 ± 2 ETDRS letters) was similar in patients receiving aflibercept compared with patients receiving bevacizumab at the end of year 1. There was also a similar percentage of patients with a mean improvement or decline in vision of 5 letters or greater (43% vs. 42% and 23% vs. 14%) in patients receiving aflibercept compared with those receiving bevacizumab, respectively (Table 4).

There were 31 eyes (27 patients) from the aflibercept group and 34 eyes (31 patients) from the bevacizumab group that were followed under the TEP/M protocol for at least 2 years (without deviations from the protocol as described for year 1). The mean interval between treatments (using a maximal interval of 6 months for patients who were weaned off treatment) remained higher in the patients receiving aflibercept compared with the patients receiving bevacizumab (17.5 ± 1.5 weeks vs. 12.0 ± 1.4 weeks; *P* = 0.010) after 24 months (Table 5). In turn, the mean number of treatments received by the end of year 2 was lower for patients receiving aflibercept than for patients receiving bevacizumab (10.2 ± 0.9 vs. 14.0 ± 0.8; *P* = 0.001). The mean change in CST (–80.9 ± 20.7 μm vs. –95.6 ± 17.1 μm; *P* = 0.752) was similar between patients receiving aflibercept and those receiving bevacizumab at the end of year 2. Compared with traditional bimonthly treatment with aflibercept (following 3 initial monthly treatments), the number of treatments using the TEP/M protocol was reduced by 27% (from 14.0 to 10.2) at 24 months (Table 5). Compared with traditional monthly treatment with bevacizumab, the number of treatments using the TEP/M protocol was reduced by 44% (from 25.0 to 14.0) at 24 months.

### Patients successfully weaned off anti-VEGF therapy using the TEP/M approach.

In the first year, 47% (33 of 70) of eyes (30 of 60 patients) receiving aflibercept were quiescent 12 weeks after their prior treatment compared with 15% (8 of 52) of eyes (8 of 46 patients) receiving bevacizumab (Supplemental Table 1; supplemental material available online with this article; https://doi.org/10.1172/JCI159125DS1). At their first 6-week monitoring visit, 90% (30 of 33) of eyes of patients receiving aflibercept remained quiescent; the other 3 eyes of 3 patients required maintenance treatment every 12 weeks. Eight of 8 eyes of 8 patients receiving bevacizumab remained quiescent at their 6-week and 18-week monitoring visits (Supplemental Table 1).

At the end of year 1, there was a trend toward more eyes of patients receiving bevacizumab (23%; 12 of 52) requiring treatment every 4 weeks compared with eyes of patients receiving aflibercept (13%; 9 of 70); this was not statistically significant (*P* = 0.066; Table 6). However, more eyes receiving bevacizumab required treatment every 6–8 weeks (44%; 23 of 52) compared with eyes receiving aflibercept (27%; 19 of 70). Similar numbers of eyes of patients receiving aflibercept or bevacizumab were extended to 10–12 weeks between treatments at the end of year 1 (Table 6). More eyes were successfully weaned off treatment (i.e., patients not requiring treatment on 3 consecutive scheduled visits and for at least 30 weeks from their last injection) with aflibercept compared with treatment with bevacizumab (43% vs. 15%; *P* < 0.0001) by the end of year 1 (Table 6). Of the eyes of patients who were followed for 2 years, 52% (16 eyes of 12 patients) were weaned off aflibercept compared with the 27% (9 eyes of 9 patients) that were weaned off bevacizumab (Table 7). Of the 16 eyes from 14 patients successfully weaned off aflibercept who were followed for a minimum of 2 years, 75% of eyes (12 of 16) remained off treatment at the end of year 2 (Supplemental Table 2). Of the 6 eyes from 6 patients successfully weaned off bevacizumab who were followed for a minimum of 2 years, 67% of eyes (4 of 6) remained off treatment for 2 years. Overall, 73% of eyes that weaned off treatment in the first year remained off treatment at the end of the second year, and 87% of eyes weaned off treatment in year 2 remained off treatment at the end of the third year (Supplemental Table 2).

To determine whether inclusion of second eyes of patients biased the outcome, we repeated our statistical analyses after excluding second eyes of patients with both eyes enrolled and found that the percentage of eyes that were weaned off treatment remained unchanged for both the aflibercept and bevacizumab groups at the 12-month time point (Supplemental Table 3). At the 24-month time point, we observed similar results for the percentage of eyes weaned off treatment for the bevacizumab group, but we observed a modest decrease (52% to 44%) in the percentage of eyes that were ultimately weaned off treatment when second eyes were excluded for the aflibercept group (Supplemental Table 3).

We next examined whether closer monitoring of the fellow eye while treating the first eye may have led to a bias in the data. We found that 10 of 60 (17%) eyes treated with aflibercept and 6 of 46 (13%) eyes treated with bevacizumab developed choroidal neovascularization (CNV) in their fellow eye over the duration of the study (Supplemental Table 4); this difference was not statistically significant (*P* = 0.428). The mean treatment interval at the end of 12 months for the fellow eye was similar to that of the first eye (Supplemental Table 5). However, the difference in the treatment interval between the first and second eye of the same patient was variable. For almost half of these patients (7 of 16; 44%) the response of the second eye was similar to that of their first eye. However, some second eyes responded better (4 of 16; 25%) while others responded worse (5 of 16; 31%). Although the number of eyes was small, half of second eyes (5 of 10) treated with aflibercept responded worse than the first eye (Supplemental Table 6); this was not the case for any of the second eyes treated with bevacizumab.

### Response of treatment-naive and reactivated CNV eyes to the TEP/M approach.

The majority of eyes of patients included in this study had newly diagnosed (treatment-naive) nvAMD. However, a subset of eyes had reactivated CNV (i.e., newly active CNV that had previously been quiescent and not received treatment for at least 1 year; Supplemental Table 7). In treatment-naive eyes, 39% (22 of 57) receiving aflibercept, compared with 16% (7 of 43) receiving bevacizumab, were successfully weaned off treatment by 12 months (Supplemental Table 8). Interestingly, for eyes with reactivated CNV, 62% (8 of 13) were successfully weaned off aflibercept while only 11% (1 of 9) were successfully weaned off bevacizumab.

### Aqueous VEGF levels were similar in patients with nvAMD treated with aflibercept or bevacizumab.

**Figure 2:**
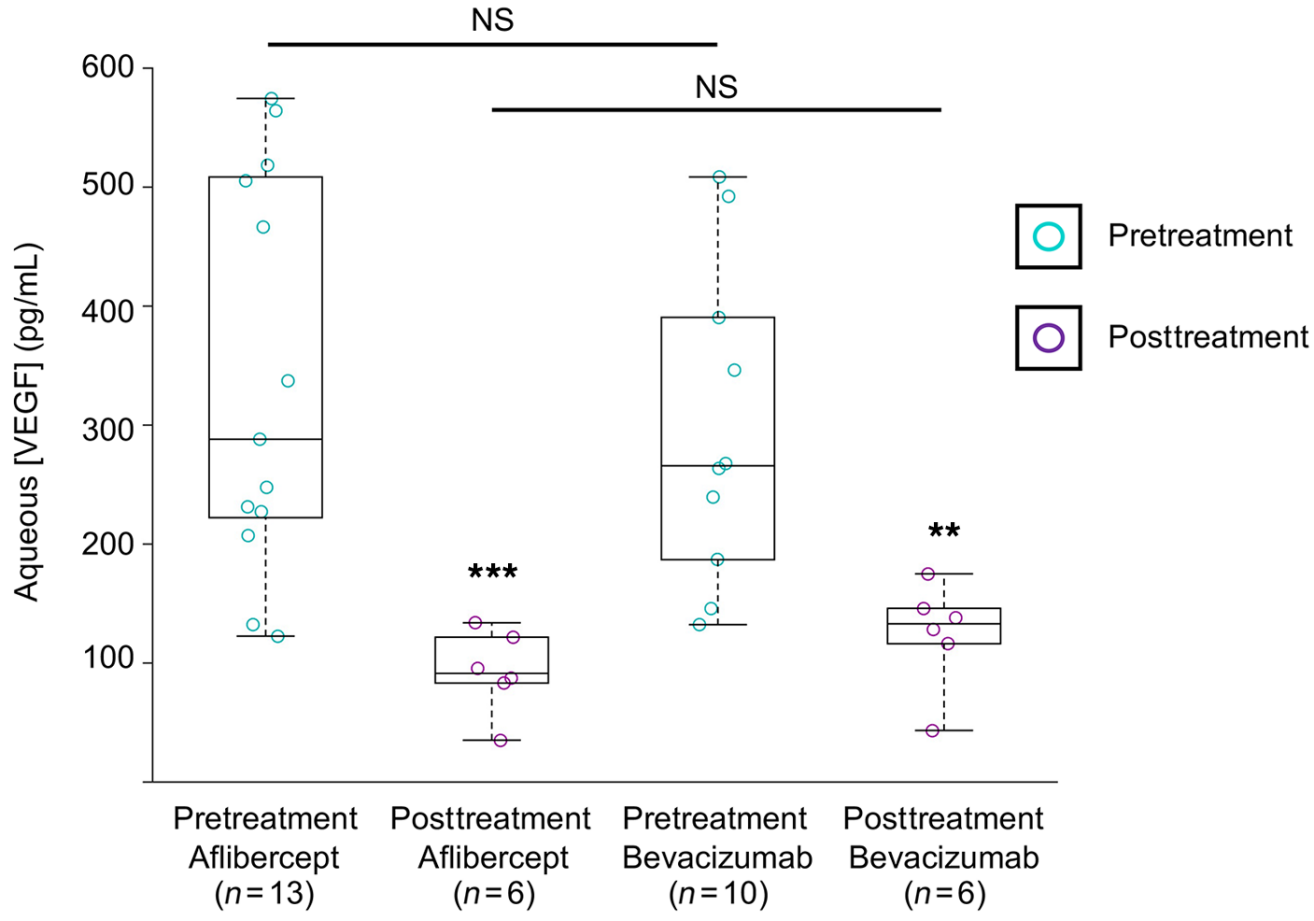
Aqueous levels of VEGF in patients on the TEP/M protocol receiving aflibercept or bevacizumab. Comparison of pretreatment and posttreatment aqueous VEGF levels for patients receiving aflibercept or bevacizumab (from subset of patients on the TEP/M protocol). Statistical analysis was performed using Graphpad Prism with the Mann-Whitney test. **P < 0.01; ***P < 0.001.

We next sought to determine whether the advantage of aflibercept over bevacizumab may be due, in part, to the fact that aflibercept may reduce VEGF levels more effectively. A subset of patients included in our study consented to provide aqueous samples on presentation, prior to initiation of treatment with anti-VEGF therapy. The concentration of VEGF in these aqueous samples was measured by ELISA in patients who were treated with aflibercept and compared with patients who received bevacizumab. Pretreatment aqueous levels of VEGF were similar in both groups (Figure 2). The posttreatment aqueous levels of VEGF within the first 3 months of initiating anti-VEGF therapy were markedly decreased in patients treated with aflibercept or bevacizumab and were also similar in both groups (Figure 2).

### Differences on OCT in response to aflibercept and bevacizumab using the TEP/M approach in patients with nvAMD.

**Figure 3:**
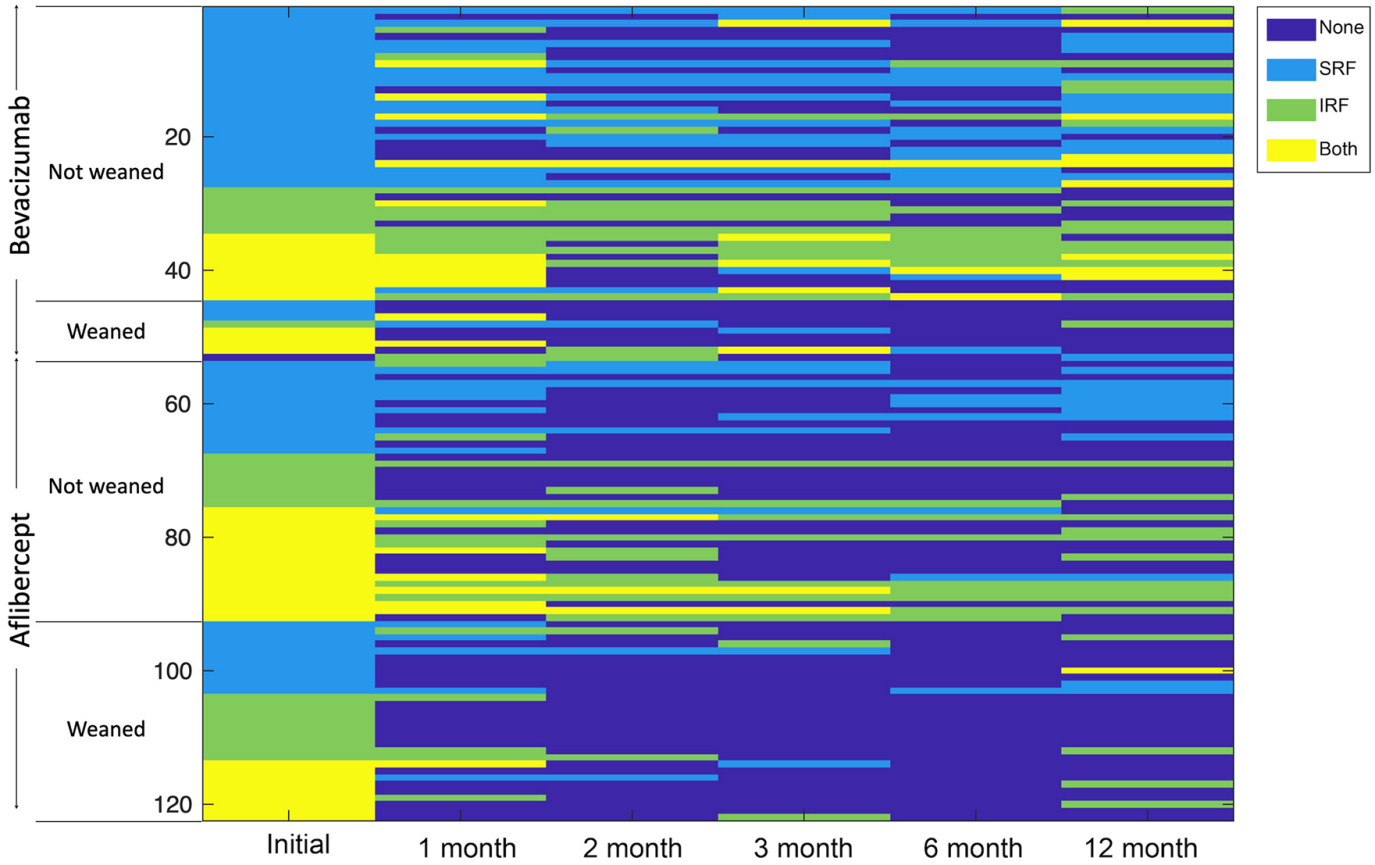
Heatmap comparing fluid over time for eyes of patients who required sustained anti-VEGF treatment and those who were successfully weaned from aflibercept or bevacizumab by 12 months. SD-OCT images were obtained from all 122 eligible eyes that underwent the TEP/M approach for at least 12 months. Presence of fluid on OCT was graded independently by two investigators for the presence of no fluid (None), subretinal fluid (SRF), intraretinal fluid (IRF), or both at the following time points: 0, 1, 2, 3, 6, and 12 months after initiation of protocol. Fluid status over time for each individual patient is graphically represented, with dark blue denoting no fluid, light blue representing SRF, light green indicating IRF, and yellow denoting both. Patients treated with aflibercept or bevacizumab were grouped into 2 categories: those not weaned (requiring sustained treatment every 4–12 weeks) and those weaned off treatment by 12 months. Within each group, patients were sorted by degree of fluid status (none < SRF < IRF < both).

We next set out to determine whether we could distinguish between patients who received aflibercept or bevacizumab based on their SD-OCT findings following treatment initiation. To this end, SD-OCT images from patients with nvAMD were graded prior to initiation of treatment, at the time of diagnosis (i.e., at presentation), and at 1, 2, 3, 6, and 12 months after initiating treatment for the presence of fluid. Each SD-OCT was classified as having no fluid, subretinal fluid (SRF), intraretinal fluid (IRF), or SRF and IRF by 2 independent masked graders; disagreements were reconciled by a third grader. Patients were then divided based on if they received aflibercept or bevacizumab and whether they could be successfully weaned from treatment (Figure 3). Interestingly, while the distribution of fluid was similar in both groups at presentation (Supplemental Table 9), 50% (35 of 70) of eyes of patients receiving aflibercept had complete resolution of fluid after their first treatment compared with only 27% (14 of 52) of eyes of patients receiving bevacizumab (Table 8). The advantage of aflibercept over bevacizumab for complete resolution of fluid peaked at 6 months but was maintained for the duration of year 1 (Table 8). Collectively, these results demonstrate a previously unappreciated advantage of aflibercept over bevacizumab for weaning patients with nvAMD off treatment.

## Discussion

Between 2013 and 2016, almost one-third of treated patients with nvAMD received aflibercept and approximately one-half received bevacizumab (1). However, direct comparisons between aflibercept and bevacizumab for the treatment of nvAMD are limited. A recent systematic review comparing the safety and efficacy of aflibercept, bevacizumab, and ranibizumab failed to find a significant difference among the 3 agents (15). While aflibercept has been reported to produce greater reduction in CST and resolution of fluid on SD-OCT in patients previously thought to be resistant to other anti-VEGF therapies, despite a reduction in the number of injections over 1 year (4, 16, 17), a meta-analysis of 28 studies showed that treatment with aflibercept did not result in a statistically significant improvement in visual acuity at 6 and 12 months after patients were switched (5). More recent studies suggest that the success of aflibercept in patients previously thought to be resistant to other anti-VEGF therapies may simply be a consequence of regression to the mean (18, 19). Collectively, these studies have raised doubts regarding the advantage of aflibercept over bevacizumab for the treatment of patients with nvAMD. Accordingly, some insurance companies are now requiring all patients with nvAMD start treatment with bevacizumab and allow for transitioning to aflibercept only in patients who fail treatment with bevacizumab.

Here, we compared aflibercept and bevacizumab using a hybrid of the as-needed PRN protocol and the personalized TAE protocol, in which we optimized the efficacy of each drug for a specific patient, while minimizing the number of treatments needed. After a mandatory 3 consecutive monthly treatments, we observed a modest advantage of aflibercept over bevacizumab for the percentage of patients with a 5 or more letter gain in vision. This was consistent with findings in prior studies, demonstrating that aflibercept may be more effective than bevacizumab when used at a similar treatment interval (3). However, at 6 months, during the dynamic TAE phase of the protocol, while determining the ideal interval between treatments for each individual patient, this advantage for aflibercept was no longer observed. Conversely, we did observe a lower rate of vision loss in patients receiving aflibercept compared with those receiving bevacizumab. This was consistent with a higher failure rate when the interval between treatments was extended for patients receiving bevacizumab compared with those receiving aflibercept (as is reflected at 6 months by the shorter treatment interval for patients receiving bevacizumab compared with those receiving aflibercept). By 1 year, as patients reach a steady state for treatment, no differences in vision or SD-OCT findings were observed between patients receiving aflibercept and those receiving bevacizumab.

The treatment interval for eyes of patients receiving aflibercept was 44% longer compared with that for eyes receiving bevacizumab (13.1 ± 0.9 weeks vs. 9.1 ± 0.8 weeks; *P* = 0.001) by the end of year 1. The advantage of aflibercept over bevacizumab for the prolonged treatment interval was maintained at the end of year 2 (17.5 ± 1.5 weeks vs. 12.0 ± 1.4 weeks; *P* = 0.010). This, in turn, resulted in a decrease in the number of injections by 27% in eyes of patients receiving aflibercept compared with eyes of patients receiving bevacizumab using the TEP/M protocol (10.2 ± 0.9 vs. 14.0 ± 0.8; *P* = 0.001) at the end of year 2. Collectively, these results demonstrate that TEP/M with aflibercept is particularly effective in reducing the number of treatments and extending the interval between visits for patients with nvAMD.

These results have important implications for the management of patients with nvAMD. We confirmed that using aflibercept can result in a longer treatment interval and fewer injections using a TAE approach compared with bevacizumab. Conversely, while the number of treatments was slightly higher for the bevacizumab group, the final CST and the final visual acuity were similar at 1 year regardless of the choice of drug. These results demonstrate that more frequent treatment with bevacizumab may be equivalent to treatment with aflibercept. Despite more required treatments, this suggests that TAE with bevacizumab, which is a fraction of the cost of aflibercept, may still be more cost-effective than TAE with aflibercept (20).

However, we further observed that 43% of eyes of patients with nvAMD receiving aflibercept entered a treatment pause within 1 year of treatment initiation compared with only 15% of eyes of patients receiving bevacizumab. By the end of year 2, approximately half (52%) of eyes of patients with nvAMD treated with aflibercept entered a treatment pause compared with one-quarter (27%) of eyes of patients treated with bevacizumab. The potential long-term cost-savings for patients with nvAMD successfully weaned off treatment — even temporarily — raises questions as to the cost-effectiveness of bevacizumab compared with aflibercept and have implications for the current management of these patients. The advantage of aflibercept over bevacizumab in weaning patients off treatment was particularly notable in patients with nvAMD with reactivated CNV (62% vs. 11%). This suggests an additional benefit of aflibercept over bevacizumab in the treatment of patients with reactivated CNV.

Of note, it has previously been reported that second eyes fare better than first eyes in patients with nvAMD (21). However, we did not observe this advantage for aflibercept in our study. Of patients who developed active CNV in both eyes, 5 of 10 (50%) patients treated with aflibercept required more frequent treatments in their second eye compared with their first eye. Conversely, 0 of 6 patients treated with bevacizumab required a shorter treatment interval in their second eye. While the sample size is small, it is tempting to speculate that this may be a consequence of differences in tachyphylaxis between these 2 drugs (22).

The observation that the early response to treatment with aflibercept was significantly greater than the response to bevacizumab, and that this correlated with the improved efficacy of aflibercept over bevacizumab at weaning patients off treatment, suggests that there may be an unexpected long-term advantage to starting patients on treatment with aflibercept. If the early treatment phase using the TEP/M approach influences whether the CNV enters quiescence, during which time a patient can safely be followed while their treatment is paused, initiating treatment with bevacizumab may ultimately limit the efficacy of the TEP/M approach to wean patients off therapy. Indeed, after 2 years of treatment, patients treated with bevacizumab were still half as likely to be weaned off treatment compared with patients treated with aflibercept. This raises questions about the long-term impact of requiring patients to initiate treatment with bevacizumab and switch to aflibercept only after patients fail treatment with bevacizumab. Ultimately, the advantages of aflibercept over bevacizumab in terms of its duration of action (and, in turn, fewer treatments/visits) and improved efficacy at weaning patients off anti-VEGF therapy needs to be balanced by the substantial cost differences between the 2 drugs. Future studies modeling the cost-effectiveness of each medication will need to account for these factors as well as the theoretical benefits to patients who are spared frequent intraocular injections.

We speculate that early treatment strategies that more effectively quench the angiogenic drive in nvAMD eyes may be necessary to achieve CNV quiescence; this emphasizes the importance of early detection and treatment of CNV lesions. We postulate that the advantage to patients with nvAMD who receive aflibercept over bevacizumab in successfully weaning off treatment may be attributed to the increased efficacy of aflibercept in promoting CNV quiescence. It is important to note that, in patients with nvAMD with quiescent CNV, the CNV likely remains present but is inactive. Close monitoring is therefore essential for the detection of CNV reactivation, at which point treatment needs to be resumed. Whether quarterly monitoring by a vision care provider (as was performed in this study) is sufficient for the early detection of CNV reactivation, or additional measures (e.g., home monitoring; ref. 23) would be of benefit, remains unanswered.

What also remains unclear is why one therapy would be more effective at weaning patients with nvAMD off treatment than another therapy if both target the same protein. Aflibercept and bevacizumab effectively bind to (and neutralize) VEGF-A. While we did not observe a difference in the aqueous levels of VEGF-A following treatment with aflibercept compared with bevacizumab, we cannot state that this reflects the efficacy of the 2 drugs at neutralizing VEGF-A in the retina/choroid. However, in addition to targeting VEGF-A, aflibercept also binds to other members of the VEGF family, including VEGF-B/C/D and placental growth factor, which have collectively been implicated in the promotion of pathological angiogenesis, vascular leakage, neurodegeneration, and inflammation, independent of VEGF (24). We recently reported that aqueous levels of another vasoactive mediator, angiopoietin-like 4, correlate with the response of patients with nvAMD to anti-VEGF therapies (25). If the advantage of aflibercept over bevacizumab is because aflibercept can bind to vasoactive mediator(s) in addition to VEGF-A, new therapies that target more than one vasoactive mediator, such as the recently FDA-approved bispecific (anti-VEGF/anti-ANGPT2) agent faricimab (26, 27), may be the most-effective approach for safely achieving CNV quiescence. We speculate that the ability to extend the treatment interval with faricimab to 4 months in approximately one-half of patients with nvAMD in the TENAYA and LUCERNE trials (28) may be due to CNV quiescence, similar to what we propose also occurs in the patients with nvAMD treated with aflibercept who enter a treatment pause. We, therefore, propose that the induction of CNV quiescence (as assessed by the ability to enter a treatment pause) should be included as an arm in future clinical studies assessing current and new therapies for nvAMD.

The observation that 43% of eyes of patients with nvAMD treated with aflibercept could achieve a treatment pause within 1 year (and half of eyes by the end of the second year) further suggests many patients with nvAMD may not require — or benefit from — the anticipated introduction of second-generation, longer-acting anti-VEGF therapies (29) or alternative delivery techniques (e.g., surgical implantation of an anti-VEGF reservoir; ref. 30). This is particularly important given emerging concerns regarding potentially adverse consequences of long-term suppression of VEGF in the eyes of patients with AMD (31). Collectively, these observations may influence clinical consideration of how to identify the best candidates for newer, longer-acting anti-VEGF therapies.

One of the major criticisms raised about protocols in which anti-VEGF therapy is paused is that this approach may expose patients with nvAMD to a recurrence of CNV (and vision loss), which could otherwise have been prevented if the patient received continued (i.e., maintenance) treatment. Although we did not observe an increased risk of vision loss for patients in whom treatment was paused compared with the patients who were treated every 8–12 weeks (11), this was not directly assessed in our patients. Several prior studies have explored the risks of pausing treatment in patients with nvAMD, particularly the concern for permanent vision loss in patients who develop recurrence of CNV and subsequently require retreatment. A recent study looking at the risks of treatment suspension found that up to 41% of eyes showed recurrent disease within 1 year, increasing up to 79% by year 5 of holding treatment (32). The authors noted that patients who experienced recurrence of CNV lost the visual improvements that they gained while undergoing treatment and were only partially able to regain this vision once treatment was resumed. While it is tempting to speculate from these observations that withholding treatment directly contributes to vision loss (33), this study lacked a control population who received continued maintenance therapy for comparison. In a separate study, investigators looked at treatment cessation under a treat-extend-stop regimen and found that, while patients who developed recurrence did initially lose vision compared with vision at the time of treatment cessation, this vision loss was reversible; retreatment allowed patients to recover the vision that was lost, ultimately resulting in noninferior visual outcomes compared with vision at the time point when treatment was initially held (13). Similarly, a third study in which treatment was held after successful resolution of disease with aflibercept found that the vision in patients with recurrent CNV 6 months after holding treatment did not differ from those who remained quiescent (34). The authors concluded that continuing injections in these patients would result in overtreatment, as up 70% of patients were able to achieve a dry macula after 1 year of treatment, with greater than 55% of these patients remaining disease free at the 2-year mark. These results are consistent with those from a post-hoc analysis of the CATT trial, which noted that a subset of patients can maintain good visual acuity, despite holding treatment for 3 years after an initial treatment period (35).

The importance of including a control arm with patients receiving maintenance therapy in studies examining the potential risks and benefits of holding treatment is emphasized by recent reports that continuing treatment with anti-VEGF therapy after achieving functional and morphological stability does not prevent recurrence of CNV. Two recent studies both concluded that patients continue to have a lifetime risk of recurrence that accumulates with each additional year of treatment, despite ongoing maintenance therapy with anti-VEGF (36, 37). Indeed, maintenance anti-VEGF therapy has not proven to be preventative of vision loss in all patients; a subset of patients continue to experience vision loss despite treatment at regular intervals in each of the major prospective clinical trials. It was noted in a post-hoc analysis of the CATT trial that a small percentage of eyes developed sustained vision loss despite ongoing treatment with anti-VEGF therapy (38). The VIEW 1/2 and LUCAS trials also reported a subset of patients who lost vision despite continued treatment during the trials (2, 39). It is therefore not clear whether vision loss observed in patients with nvAMD undergoing a treatment pause would have occurred even if maintenance treatment was continued.

Collectively, these data do not support the hypothesis that maintenance therapy more effectively prevents either recurrent CNV or the vision loss that accompanies recurrence compared with a PRN approach once CNV activity is no longer detectable. This assumption is further weakened by the observation from prospective clinical studies that PRN therapy for nvAMD, which also includes a treatment pause, has been shown to be noninferior to continued treatment (7, 8). Conversely, a recent study demonstrating that prophylactic quarterly injections with anti-VEGF therapy failed to reduce the conversion of patients with AMD from nonneovascular to neovascular undermines the assumption that sustained VEGF suppression is sufficient to prevent CNV (40). Given the concern that overtreatment may unnecessarily expose patients to the known risk of intravitreal injections (e.g., retinal tears, retinal detachment, and endophthalmitis), as well as the theoretical risk of sustained VEGF suppression (31), whether (indefinite) maintenance therapy is a safer or more effective approach than protocols that include a treatment pause remains an unanswered question.

The limitations of our study include that it is a retrospective cohort study. The study design introduced biases that need to be weighed when assessing outcomes measured. However, a recent review summarizing the results of methodological reviews that compared the outcomes of observational studies (including retrospective cohorts) with randomized trials addressing the same question demonstrated that there is little evidence for significant estimate differences between observational studies and randomized control trials, regardless of the specific observational study design (41). The authors concluded that it is therefore important to consider other factors that may influence study outcomes. In this regard, additional limitations include that there were a limited number of patients and a follow up of 1 year (with a subset of patients followed for 2 to 3 years). The stringent inclusion criteria resulted in the exclusion of a number of patients from those who were initially screened (the majority of whom did not consent or adhere to the TEP/M protocol), but this was done to minimize confounding variables and increase the generalizability of our findings.

Another important limitation of this study is that the study arms were not randomized. However, the choice between treatment with bevacizumab and aflibercept was made by the patient, rather than the treating physician (14). We previously reported that in patients who were given the choice between a more cost-effective drug (i.e., bevacizumab) and the most effective drug (i.e., aflibercept) for their own eye care, altruism was the strongest predictor for patients selecting bevacizumab over aflibercept (14). Patients were only included in this study if their insurance covered the cost of both drugs, thereby minimizing the influence of out-of-pocket costs on the patient’s choice. Accordingly, the 2 patient groups presented with similar baseline characteristics; we did not observe any difference in vision, clinical exam, or SD-OCT findings between the 2 treatment groups prior to initiation of treatment. Nonetheless, these limitations must be considered when interpreting the results presented here.

To our knowledge, there has not been any prior published report — retrospective or prospective — comparing the 2 most frequently used anti-VEGF therapies, aflibercept and bevacizumab, in a head-to-head comparison using a protocol designed to wean patients with nvAMD off treatment. A prospective randomized clinical trial assessing the safety and efficacy of the TEP/M approach compared with TAE with maintenance therapy or monthly/bimonthly treatment will ultimately be needed to assess the risks and benefits of each of these approaches. Studies incorporating newer therapies that target multiple vasoactive mediators (e.g., faricimab) may further improve our understanding of the factors that influence the ability for therapies to promote CNV quiescence. Until then, considerations for current management of patients with nvAMD should consider the potential advantage of more rapid resolution of fluid and, in turn, greater success at weaning patients off therapy using the TEP/M approach with anti-VEGF therapies.

## Methods

### Patients.

Insured patients at a satellite office (primary cohort) or a hospital-based clinic (secondary cohort) of a tertiary care center with a diagnosis of nvAMD were treated with bevacizumab or aflibercept (collectively, anti-VEGF therapy). Please see Supplemental Methods for detailed description of patient inclusion and exclusion criteria.

### Selection of aflibercept or bevacizumab for patients.

Please see Supplemental Methods for a detailed description of patient selection between aflibercept or bevacizumab. Briefly, patients were asked to choose between the 2 drugs following a detailed description of their costs and benefits. Clinical presentation did not influence this decision. Moreover, this decision was not influenced by the treating physician. This protocol for drug selection by patient has been previously reported (14).

### TEP/M protocol.

Please see Supplemental Methods for a detailed description of the TEP/M protocol.

### Grading fluid status over time.

SD-OCT images were obtained and analyzed from all eligible patients enrolled in the study who underwent the TEP/M approach for at least 12 months. SD-OCT images were graded after initiation of protocol for the presence of fluid by 2 independent and masked graders at the following time points: 0, 1, 2, 3, 6, and 12 months; OCTs performed at the time points closest to 6 and 12 months (i.e., within 4 weeks of the 6-month time point and 6 weeks of the 12-month time point from treatment initiation) were included in this analysis. Each image was classified as having no fluid, SRF, IRF, or both. Differences in image grading between the 2 blinded graders were reconciled, and if an agreement could not be reached, a third investigator cast the tie-breaker vote. The location of fluid for each patient and at each time point was graphically represented in a table with different color cells representing no fluid, SRF, IRF, or both. Patients were grouped based on frequency of treatment at 12 months to compare those who were able to successfully pause treatment with those who were unable to reach treatment pause.

### Aqueous samples.

Aqueous samples (0.1–0.2 mL) were collected via limbal paracentesis using a 30-gauge needle attached to a tuberculin syringe from consenting patients at the Wilmer Eye Institute immediately after performing intravitreal injection for active CNV. Aqueous samples were immediately processed and stored at –80°C prior to analysis.

### ELISA.

Human VEGF ELISA kits (Duoset, DY293B) were purchased from R&D Systems. Aqueous samples were analyzed for VEGF (10 μL of aqueous diluted 1:10) using ELISAs, which were performed according to the manufacturer’s protocols. All ELISAs were performed in duplicate (VEGF), and quantitation was performed using the standard curve constructed with the standards included in the kit.

### Statistics.

Categorical variables were presented as percentages and compared using the 2-sided χ^2^ test, with significance set at *P* < 0.05. Patient best corrected visual acuities were converted from Snellen visual acuity into a logarithm of the minimum angle of resolution (logMAR) score for statistical analysis using the following formula for conversion: logMAR = –1 × log_10_(snellen fraction) (42). This score was further adjusted by adding or subtracting 0.02 for each letter correctly or incorrectly identified on the previous or next line. LogMAR was then converted into approximate ETDRS scores using a standardized table to determine mean gains or losses of letters at 3, 6, 12, and 24 months compared with initial vision prior to treatment initiation. Data for continuous variables were recorded as mean ± SEM. Assuming nonparametric, unpaired, 2-tailed, Mann-Whitney test analysis, with significance set at *P* < 0.05, was used to compare mean data points in this study. All analyses were performed using GraphPad Prism 8 software.

### Study approval.

Institutional review board approval from the Johns Hopkins University School of Medicine was obtained for all patient samples, including OCT images and aqueous samples, used in this HIPAA-compliant study. Patients provided voluntary, written consent and were not provided a stipend.

## Author contributions

A Sodhi was the primary contributor to the research design. XC, JCS, TPP, ZY, CG, DM, A Sopeyin, and A Sodhi were responsible for research execution. XC, JCS, TPP, ZY, CG, and DM were responsible for data acquisition. XC, JCS, TPP, CG, SM, and A Sodhi were the primary contributors to data analysis and interpretation. A Sodhi prepared the manuscript, with revisions provided by XC, TPP, and SM. A Sodhi had full access to all the data in the study and takes responsibility for the integrity of the data and the accuracy of the data analysis

## Supplementary Material

Supplemental data

ICMJE disclosure forms

## Figures and Tables

**Table 8:**
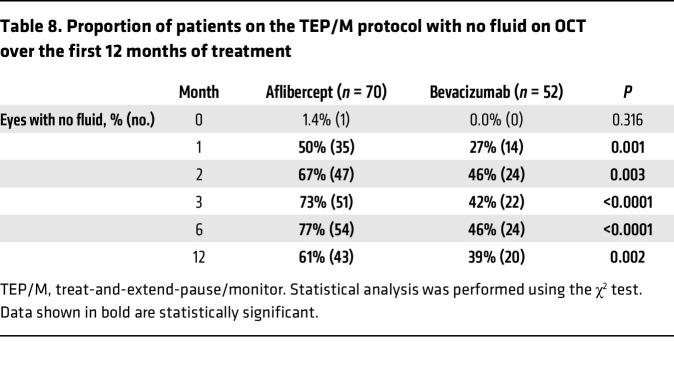
Proportion of patients on the TEP/M protocol with no fluid on OCT over the first 12 months of treatment

**Table 7:**
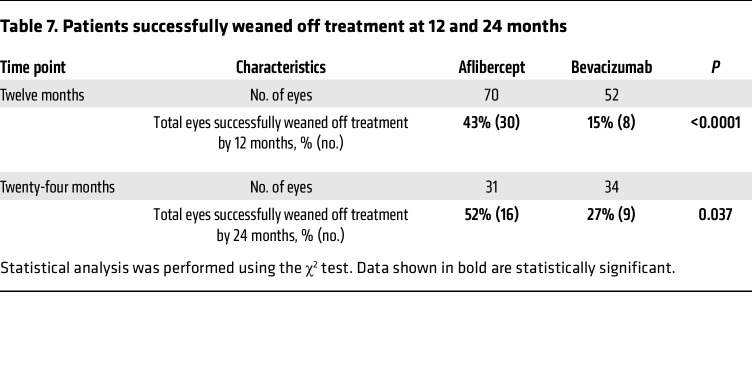
Patients successfully weaned off treatment at 12 and 24 months

**Table 6:**
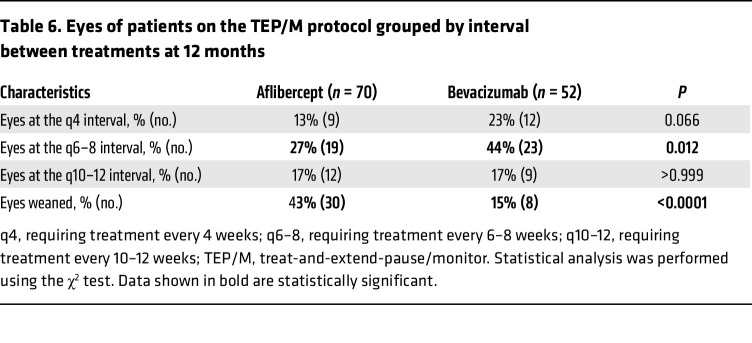
Eyes of patients on the TEP/M protocol grouped by interval between treatments at 12 months

**Table 5:**
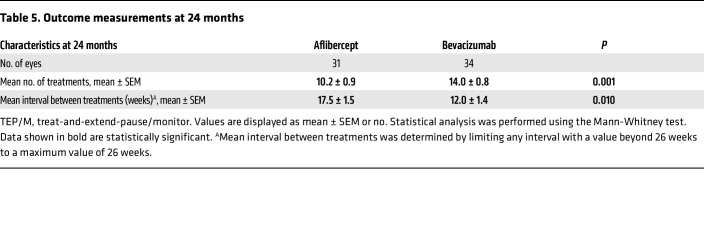
Outcome measurements at 24 months

**Table 4:**
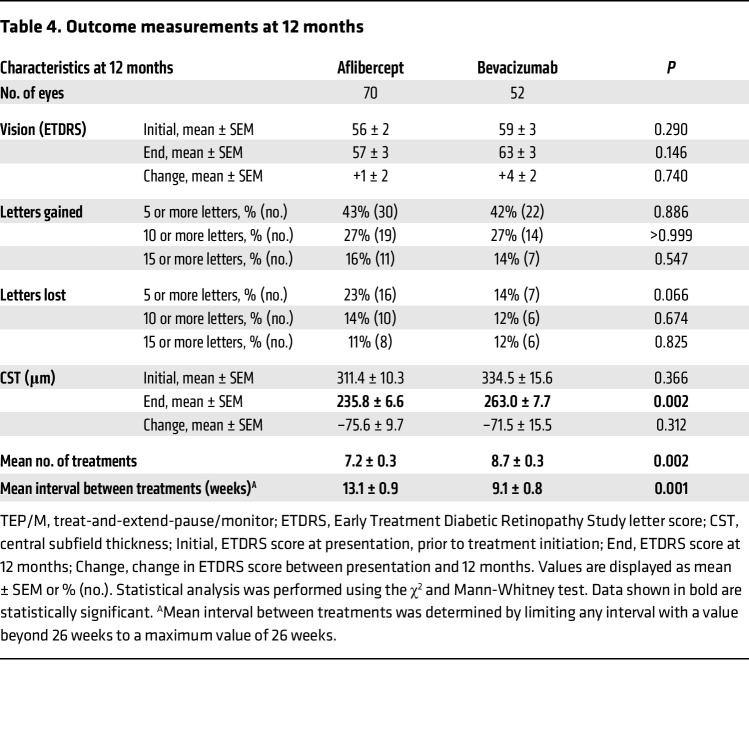
Outcome measurements at 12 months

**Table 3:**
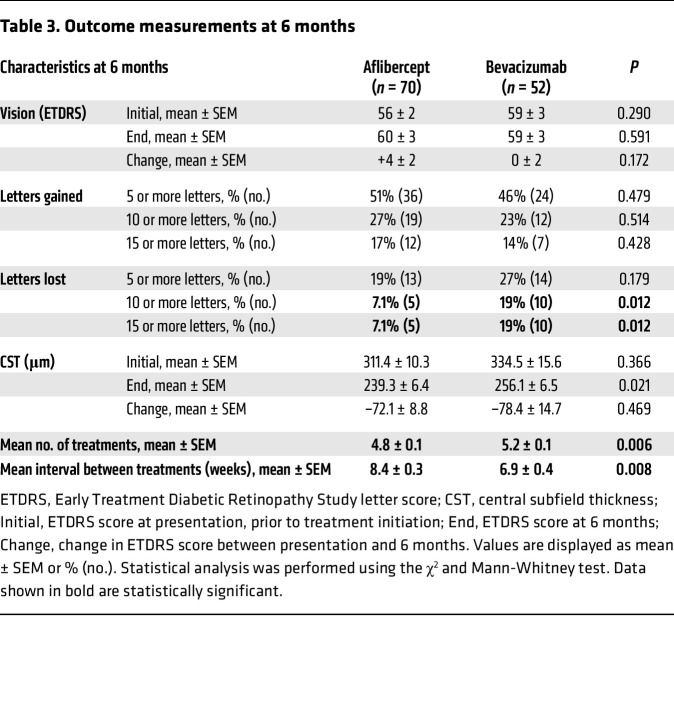
Outcome measurements at 6 months

**Table 2:**
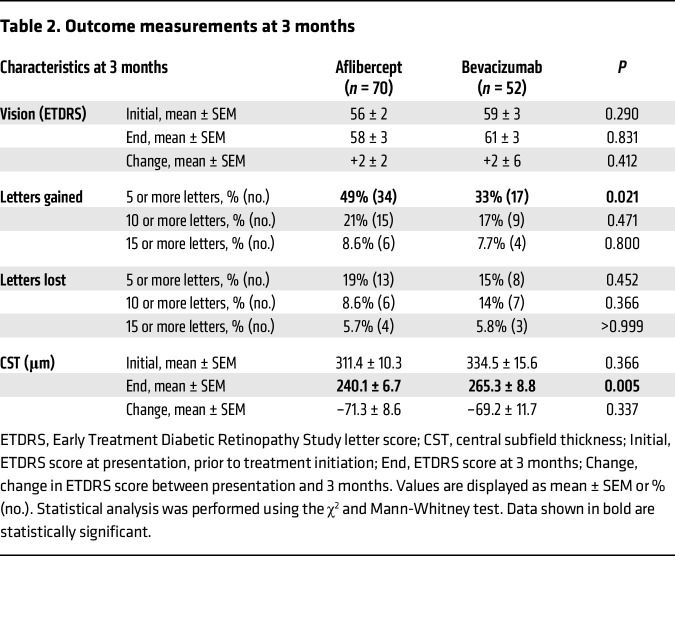
Outcome measurements at 3 months

**Table 1:**
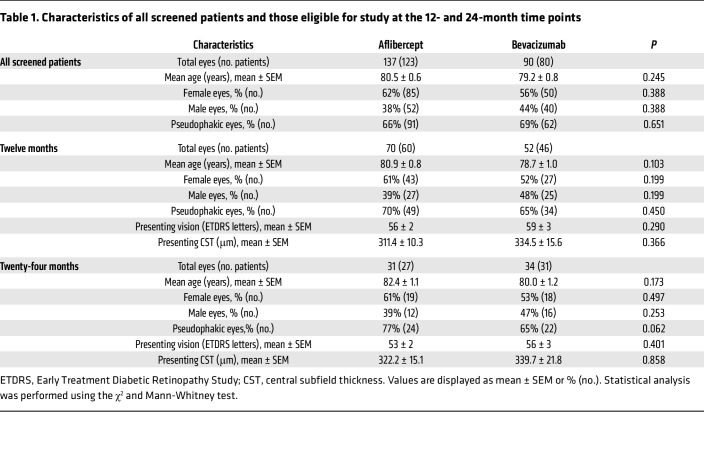
Characteristics of all screened patients and those eligible for study at the 12- and 24-month time points

## Version 1 11/22/2022

In-Press Preview

## Version 2 01/17/2023

Electronic publication
