# Supplementary material for: Aflibercept more effectively weans patients with neovascular age-related macular degeneration off therapy compared with bevacizumab
Source: J Clin Invest. 2023 Jan 17;133(2):e159125. doi: 10.1172/JCI159125 (PMC9843049; doi:10.1172/JCI159125)
Supplement: Supplemental data [file jci-133-159125-s121.pdf]

Supplemental Methods

Inclusion and exclusion criteria

Inclusion criteria included a new diagnosis of nvAMD [diagnosed on clinical exam and Spectral Domain Optical Coherence Tomography (SD-OCT) and confirmed by fluorescein angiography] or newly active nvAMD (with an absence of activity or treatment for at least 1 year and a presenting vision of 20/400 or better) who were followed after initiating treatment without interruption for at least 1 year. All patients included in this study agreed to participate in the "treat-and-extend-pause/monitor" (TEP/M) approach (see below). Exclusion criteria included an underlying ischemic retinal disease at the time of diagnosis (e.g., diabetic retinopathy, retinal vein occlusion, sickle cell retinopathy), other forms of choroidal neovascularization (e.g., presumed ocular histoplasmosis, polypoidal choroidopathy, etc.), post-surgical cystoid macular edema, active ocular inflammation, or a recent (within 1 year) history of intraocular steroid treatment. Patients in whom treatment was withheld after being determined to be "futile" (i.e., the presence of atrophy or a disciform scar involving the foveal center at presentation which prevented an improvement in vision despite an adequate response to treatment of fluid) were excluded from the study. Patients who had a delayed return for a scheduled treatment by more than 2 weeks once in the first 3 months or twice in the first year were also excluded from the study.

Selection of aflibercept or bevacizumab for patients.

Patients were given a choice between two "anti-VEGF" medications: a) bevacizumab, which is not FDA-approved for use in the eye, but is compounded in the hospital pharmacy from an FDA-approved cancer therapy, and costs approximately $100/treatment (based on the Medicare allowable charge); or b) aflibercept, which is FDA-approved for use in the eye, costs

approximately $2,000/treatment (based on the Medicare allowable charge), and has been reported to be more effective than bevacizumab for the treatment of ocular neovascular disease in some settings. The patients were also informed of their share of the cost for each medication based on their insurance coverage; patients included in this study were informed that their insurance would cover the cost of both drugs. The patient was then asked to choose which drug they preferred for their treatment. Importantly, this decision was not influenced by the treating physician {Malik, 2021 #126}.

Treat-and-Extend-Pause/Monitor (TEP/M) Protocol

All patients included in this study underwent a TEP/M approach in which patients receiving anti-VEGF therapy underwent 3 consecutive monthly injections followed by a TAE approach in which the interval between treatments was extended by 2 weeks at each visit in the absence (or near absence) of fluid, hemorrhage, or a decline in vision. Vision, clinical exam, and SD-OCT were obtained at all scheduled clinic visits. Return of fluid or a decline in vision resulted in a reduction of the interval between visits by 2-4 weeks; new hemorrhage would result in a decrease in the interval between visits back to 1 month. Stable patients extended to 12 weeks had their treatment held (paused), at which point the patients entered the "PRN phase" of treatment. After cessation of treatment, patients returned in 6 weeks (18 weeks from most recent treatment). If the patient remained stable at the 6-week follow up visit, treatment was held again, and the patient was followed every 12 weeks during the monitoring phase of the approach. A decline in vision, or a return in fluid or hemorrhage at subsequent visits would result in resuming treatment. Patients who failed an extension would be given another opportunity to have the interval between visits extended once they were stable for at least 3 consecutive visits. Patients were defined as having

been successfully paused from treatment if they did not require treatment on 3 consecutive scheduled visits following treatment cessation (i.e., at least 12 + 6 + 12 weeks; or 30 weeks total). Follow up for patients successfully paused from anti-VEGF therapy therefore required a minimum of 30 weeks beyond their last injection.

Supplemental Table 1. Success of weaning patients who achieved quiescence at 12 weeks after their last treatment.

	**Aflibercept n = 70**	**Bevacizumab n = 52**	***P***
Percent of eyes who achieved quiescence 12 weeks after their last treatment within 1 year (no.)	**47% (33)**	**15% (8)**	**<0.0001**
Percent of eyes who remained quiescent 18 weeks after their last treatment (no.)	**43% (30)**	**15% (8)**	**<0.0001**
Percent of eyes who remained quiescent 30 weeks after their last treatment (no.)	**43% (30)**	**15% (8)**	**<0.0001**

Abbreviations: No., number. Statistical analysis was performed using Chi-square. Data in bold are statistically significant.

Supplemental Table 2. Recurrence rate at 24 and 36 months.

	24 months			36 months		
**Characteristics**	**Aflibercept**	**Bevacizumab**	***P***	**Aflibercept**	**Bevacizumab**	***P***
Number of eyes weaned off treatment from previous year	16	6	-	10	5	-
Eyes with recurrent activity in 12 months following weaning - % (no.)	25% (4)	33% (2)	0.697	10% (1)	20% (1)	0.589

Abbreviation: No., number. Statistical analysis was performed using Chi-square. Data in bold are statistically significant.

Supplemental Table 3. Eyes of patients successfully weaned off treatment at 12 and 24 months (using only the first treated eye of patients who received injections in both eyes).

**Time Point**	**Characteristics**	**Aflibercept**	**Bevacizumab**	***P***
12 months	Number of eyes	60	46	
	Total eyes successfully weaned off treatment by 12 months - % (no.)	**42% (25)**	**15% (7)**	**<0.0001**
24 months	Number of eyes	27	31	
	Total eyes successfully weaned off treatment by 24 months - % (no.)	**44% (12)**	**26% (8)**	**0.008**

Abbreviation: No., number. Statistical analysis was performed using Chi-square. Data in bold are statistically significant.

Supplementary Table 4. Mean number of months between initiating treatment for nvAMD in the 1st and 2nd eye in patients who had both eyes enrolled in the TEP/M study.

**Characteristics**	**Aflibercept**	**Bevacizumab**	***P***
Number (and percent) of patients with both eyes enrolled	10 (17%)	6 (13%)	0.428
Mean months between the 1st injection dates in the 1st and 2nd eye	4.6 ± 2.3	12.4 ± 7.0*	0.090

Abbreviation: No., number; nvAMD, neovascular age-related macular degeneration; TEP/M, treat and extend pause/monitor. Values displayed as mean ± standard error of the mean. Statistical analysis was performed using Chi-square and Mann-Whitney test. Data in bold are statistically significant.

*One patient in the bevacizumab group had a difference of 46 months between initiating injections in the 1st and 2nd eye, therefore skewing the distribution. Upon exclusion of this patient, the mean interval (and p-value) for bevacizumab is 5.5 ± 1.9 (p = 0.197)

Supplementary Table 5. Comparison of mean treatment interval between the 1st eye and 2nd eye in patients who had both eyes enrolled in the TEP/M study at 12 months after initiation of therapy.

**Characteristics**	**1st Eye**	**2nd Eye**	***P***
Aflibercept mean treatment interval 12 months after initiation of therapy (months; n=10)	18.8 ± 2.3	15.2 ± 2.5	0.318
Bevacizumab mean treatment interval 12 months after initiation of therapy (months; n=6)	8.0 ± 1.4	9.7 ± 1.1	0.426

Abbreviation: n, number of patients with both eyes enrolled; TEP/M, treat and extend pause/monitor. Values displayed as mean ± standard error of the mean. Statistical analysis was performed using Mann-Whitney test. Data in bold are statistically significant.

Supplemental Table 6. Treatment interval difference between the 2nd and 1st eyes at 12 months.

Characteristic	Aflibercept n = 10	Bevacizumab n = 6	*P*
Percent (and number) of 2nd eyes in which the mean treatment interval was similar to the same patient's 1st eye (0 ± 2 weeks)	**30% (3/10)**	**67% (4/6)**	**<0.0001**
Percent (and number) of 2nd eyes in which the mean treatment interval was 4 weeks or more longer than that of the same patient's 1st eye	**20% (2/10)**	**33% (2/6)**	**0.037**
Percent (and number) of 2nd eyes in which the mean treatment interval was 4 weeks or more shorter than that of the same patient's 1st eye	**50% (5/10)**	**0% (0/6)**	**<0.0001**

Abbreviation: n, number of patients with both eyes enrolled. Statistical analysis was performed using Chi-square. Data in bold are statistically significant.

Supplemental Table 7. Distribution of eyes of TEP/M patients with newly diagnosed vs. reactivated nvAMD.

**Characteristic**	**Aflibercept n = 70**	**Bevacizumab n = 52**	***P***
Percent of eyes with newly diagnosed nvAMD (no.)	81% (57)	82% (43)	0.856
Percent of eyes with reactivated nvAMD (no.)	19% (13)	17% (9)	0.713

Abbreviation: No., number; n, sample size; TEP/M, treat and extend pause/monitor; nvAMD, neovascular age-related macular degeneration. Statistical analysis was performed using Chi-square. Data in bold are statistically significant.

Supplemental Table 8. Percentage of eyes successfully weaned off treatment at 12 months in patients with newly diagnosed vs. reactivated nvAMD.

Drug	Characteristics	Newly Diagnosed	Reactivated	*P*
Aflibercept	Number of eyes	57	13	
	Percentage of eyes successfully weaned off treatment by 12 months - % (no.)	**39% (22)**	**62% (8)**	**0.001**
Bevacizumab	Number of eyes	43	9	
	Percentage of eyes successfully weaned off treatment by 12 months - % (no.)	16% (7)	11% (1)	0.301

Abbreviation: No., number; TEP/M, treat and extend pause/monitor; nvAMD, neovascular age-related macular degeneration. Statistical analysis was performed using Chi-square. Data in bold are statistically significant.

Supplemental Table 9. Proportion of patients in TEP/M protocol with no fluid, IRF, SRF, or both on OCT at presentation.

	Aflibercept<br>n = 70	Bevacizumab<br>n = 52	*P*
Percent of eyes with no fluid (no.)	1.4% (1)	0.0% (0)	0.316
Percent of eyes with SRF (no.)	**36% (25)**	**58% (30)**	**0.002**
Percent of eyes with IRF (no.)	26% (18)	15% (8)	0.054
Percent of eyes with both (no.)	37% (26)	27% (14)	0.130

Abbreviations: No., number; n, sample size; TEP/M, treat and extend pause/monitor; IRF, intraretinal fluid; and SRF, subretinal fluid. Statistical analysis was performed using Chi-square. Data in bold are statistically significant.
